# Dysfunctional endothelial progenitor cells in patients with Hodgkin’s lymphoma in complete remission

**DOI:** 10.1002/cam4.1914

**Published:** 2018-12-13

**Authors:** Maya Wiessman, Dorit Leshem, Moshe Yeshurun, Hagai Yavin, Zaza Iakobishvilli, Pia Raanani, Ran Kornowski, Eli I. Lev, Mordehay Vaturi

**Affiliations:** ^1^ Department of Medicine D Rabin Medical Center – Beilinson Hospital Petach Tikva Israel; ^2^ Sackler Faculty of Medicine Tel Aviv University Tel Aviv Israel; ^3^ Felsenstein Medical Research Center Petach Tikva Israel; ^4^ Institute of Hematology, Davidoff Cancer Center Rabin Medical Center – Beilinson Hospital Petach Tikva Israel; ^5^ Department of Cardiology Rabin Medical Center – Beilinson Hospital Petach Tikva Israel; ^6^ Department of Cardiology Assuta Samson Ashdod Medical Center Ashdod Israel; ^7^ Faculty of Health Sciences Ben Gurion University of the Negev Beer Sheva Israel

**Keywords:** cardiovascular disease, endothelial progenitor cells, Hodgkin’s lymphoma

## Abstract

**Background:**

Patients with a history of Hodgkin's lymphoma (HL) are at increased long‐term risk of cardiovascular morbidity and mortality. Studies report an association between the pathophysiology of coronary artery disease (CAD) and levels of circulating endothelial progenitor cells (EPC), which play an essential role in vascular injury repair. The aim of the present study was to investigate the potential involvement of abnormal EPC level or function in the CAD risk of survivors of HL in remission.

**Methods:**

EPCs were isolated from peripheral blood samples drawn from 4 groups of patients aged 20‐50 years with no history of CAD: 17 patients with HL who had been in complete remission for at least 2 years, four newly diagnosed patients with HL before treatment, 28 patients with diabetes all attending a tertiary medical center, and 16 healthy individuals. Levels of EPC surface markers were measured by flow cytometry, and EPC function was evaluated by the number of colony‐forming units (CFUs) and MTT assay.

**Results:**

Levels of circulating CD34(+)/VEGFR2(+) and CD133(+)/VEGFR2(+) were significantly higher in the newly diagnosed untreated patients with HL compared to the patients with HL in remission (*P* = 0.03 and *P* = 0.005, respectively), in the patients in remission compared to the patients with diabetes (*P* = 0.011 and *P* < 0.001, respectively), and in the patients in remission compared to the healthy individuals (*P* = 0.08 and *P* = 0.003, respectively). The analysis of cell viability and the number of colony‐forming units in the patients with HL in remission yielded significant differences from the healthy group (*P* = 0.005 and *P < *0.001, respectively) but not from either the newly diagnosed patients with HL or the diabetic patients.

**Conclusions:**

These results suggest that patients in complete remission of HL for at least 2 years have an abnormal EPC profile characterized by high circulating levels but attenuated function.

## 1 INTRODUCTION

The equilibrium between endothelial injury and regeneration (repair) is of particular importance in cardiovascular health. Endothelial injury and dysfunction contribute to the development of atherosclerosis and its thrombotic complications and have been found to be predictive of cardiovascular events and death from cardiovascular causes.^1^, ^2^ For example, studies have shown that endothelial dysfunction is the cornerstone of cardiovascular morbidity in patients with type 2 diabetes mellitus and can be reversed by tight glycemic control.^3^, ^4^

Endothelial progenitor cells (EPCs) are derived from bone marrow and have the capacity to migrate to the peripheral circulation and differentiate into mature cells with an endothelial phenotype.^5^ EPCs play an essential role in vascular repair by promoting re‐endothelialization following injury.^1^ EPCs are primarily identified by the expression of combinations of cell‐surface antigenic markers, including CD133, CD34, and vascular endothelial growth factor receptor 2 (VEGFR‐2).^6^ Findings in experimental and clinical studies support the notion that the biology of EPCs is strongly related to the pathophysiology of CAD and that the circulating EPC count may serve as a surrogate marker of endothelial dysfunction.^7^ Levels of circulating EPCs were shown to be significantly lower in patients with chronic stable CAD and CAD risk factors (smoking, diabetes mellitus, family history of CAD, hypertension, elevated low‐density lipoprotein cholesterol) than in healthy individuals.^7^ Moreover, EPCs isolated from patients with CAD had an impaired migratory response that was inversely correlated with the number of risk factors. 5, 8

Hodgkin's lymphoma (HL) is a prototype of a curable malignant neoplasm, with 10‐year survival rates currently exceeding 80%.^9^ Consequently, the number of long‐term survivors is increasing every year.^10^ However, the treatment of HL has been associated with late adverse effects, such as increased risk of secondary hematological and solid neoplasms as well as cardiovascular complications.^11^, ^12^, ^13^, ^14^, ^15^, ^16^, ^17^, ^18^ Several studies reported an increased risk of cardiac mortality in survivors of HL after 15‐25 years of follow‐up,^11^, ^19^, ^20^, ^21^ and a recent study found a fourfold to sevenfold higher risk of CAD or heart failure after 35 or more years of completion of treatment compared to the general population.^13^ HL chemotherapy regimens are based on anthracycline which is notorious for its direct cardiotoxic effects. However, even in the absence of anthracycline cardiotoxicity, cardiovascular disease rates were relatively high in HL survivors.^13^ Patients exposed to mediastinal radiotherapy were also at increased risk of CAD as well as valvular heart disease and heart failure.^13^

Endothelial progenitor cells also play a role in tumor pathogenesis through the induction of neo‐angiogenesis.^22^ The number of circulating EPCs is related not only to the stage and degree of invasiveness of the tumor but also to its sensitivity to chemotherapy.^23^, ^24^ Data on various solid and lymphoproliferative malignancies have indicated an increase in the level of circulating EPCs.^23^, ^25^, ^26^

The aim of the present study was to investigate the level and function of circulating EPCs in patients with HL without a history of CAD who were in complete remission for at least 2 years after treatment with the standard anti‐HL regimen.

## 2 METHODS

### 2.1 Study group

The study group consisted of patients diagnosed with HL and treated with the standard chemotherapy regimens, namely, ABVD (adriamycin, bleomycin, vinblastine, and dacarbazine) or escalated BEACOPP (bleomycin, etoposide, adriamycin, cyclophosphamide, vincristine, procarbazine, and prednisone), with the addition of radiotherapy if indicated. We included only patients aged 20‐50 years who had been in complete remission for at least 2 years after completion of treatment. Exclusion criteria were the ad hoc presence of potential EPC effectors at recruitment as history of CAD or anthracycline‐associated cardiotoxicity, diabetes mellitus, anemia, renal failure (estimated GFR <50 mL/min according to the MDRD formula), pregnancy, statin therapy, and menopause. The age‐matched control groups consisted of patients with type 2 diabetes (diabetes group) and treatment‐naïve patients with newly diagnosed HL (acute HL group) attending outpatient clinics at the same medical center, and healthy subjects (medical and paramedical personnel from our medical center). We derived the data on the diabetic patients from a previously published paper by Lev et al^8^ The local Ethics Committee approved the study protocol (approval no. 0578‐15‐RMC). All patients and subjects signed an informed consent form to participate in the study.

### 2.2 Laboratory studies

A single blood sample (20 cc) was drawn from each patient and subject and delivered to our laboratory. Circulating EPC levels were quantified by measuring surface markers VEGFR‐2, CD34, and CD133 on flow cytometry. EPC function was evaluated by the number of colony‐forming units (CFUs), and cell viability in culture was evaluated with the MTT assay.

To quantify circulating EPC levels, peripheral blood mononuclear cells (PMNCs) were fractionated using Ficoll‐Hypaque density gradient centrifugation. The mononuclear cells gradient was isolated and washed with phosphate‐buffered saline after red cell lysis*. *Aliquots of PMNCs were incubated with monoclonal antibodies against VEGFR‐2 (FITC‐labeled) (R&D, Minneapolis, MN, USA), CD133 (PE‐labeled) (Miltenyi Biotec, Auburn, CA, USA), and CD34 (PE‐labeled, Miltenyi Biotec). Isotype‐identical antibodies were used as controls. After incubation, the cells were washed with phosphate‐buffered saline and analyzed with a flow cytometer (FACSCalibur, Becton Dickinson, Franklin Lakes, NJ, USA). Each analysis included 100 000 events, after selection for viability and CD‐45‐positive cells and exclusion of debris and platelets. In the next step, gated CD34‐ or CD133‐ positive cells were examined for expression of VEGFR‐2. Results are presented as the percentage of cells co‐expressing either VEGFR‐2 and CD133 or VEGFR‐2 and CD34.

To evaluate EPC function, isolated PMNCs were re‐suspended with Medium 199 (Invitrogen, Carlsbad, CA, USA) supplemented with 20% fetal calf serum (Gibco BRL Life Tech, Gaithersburg, MD, USA) and plated on 6‐well plates coated with human fibronectin at a concentration of 4*10^6^ cells per well. EPC colonies were counted using an inverted microscope 7 days after plating. An EPC colony was defined as a cluster of at least 100 flat cells surrounding a cluster of rounded cells, as previously described.^3^ A central cluster alone without associated emerging cells was not considered a colony. Colonies were counted manually in 3‐10 random microscopic fields. To confirm endothelial cell lineage, indirect immune‐staining of randomly selected colonies was performed with antibodies directed against VEGFR‐2, CD31 (Becton Dickinson), and Tie‐2 (Santa Cruz Biotechnology, Santa Cruz, CA, USA). Results were expressed as the mean number of CFUs per field.

The viability of the cultured EPC cells was evaluated with the MTT assay (Sigma, St. Louis, MO, USA). MTT (3‐[4,5‐dimethylthiazol‐2‐yl]‐2,5‐diphenyl tetrazolium bromide) measures mitochondrial activity in living cells. After 7 days of culture, 1 mg/mL MTT was added to the EPC medium culture and incubated for an additional 3‐4 h. The medium was then removed, and the cells were solubilized in isopropanol. Mitochondrial dehydrogenases of viable cells cleave the tetrazolium ring, yielding purple MTT crystals, which can be dissolved in isopropanol. The amount of the dye released from the cells was measured with a spectrophotometer at 570 nm and subtracted background at 690 nm. An increase in the number of viable cells results in an increase in the amount of MTT formed and, therefore, in absorbance. Therefore, optical density is directly correlated with viable cell quantity.

### 2.3 Statistics

Parameters are presented as mean and standard deviation. Intra‐group comparisons were performed by two‐factor analysis of variance (for group‐ and time‐dependence). Normally distributed continuous variables were compared by t tests, and non‐normally distributed variables by Mann‐Whitney U test**.** Categorical variables were compared using chi‐square tests. *P* < 0.05 was considered statistically significant. Analyses were performed using SPSS version 23 statistical software (SPSS Inc, Chicago, IL).

## 3 RESULTS

The cohort included 17 patients with HL in remission, four patients with newly diagnosed, as yet untreated, HL, and 16 healthy subjects matched by age and sex to the patients in remission. These three groups were recruited between February 2017 and May 2018. A fourth group of 28 patients with diabetes had been recruited for another EPC study^8^ and were characterized by older age and a more robust risk profile for atherosclerotic cardiovascular disease compared to patients without diabetes.

**Figure 1:**
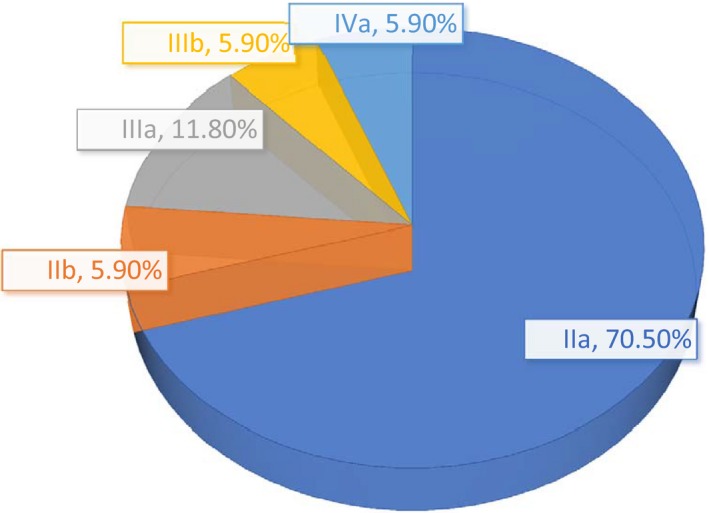
Stage of HL at diagnosis in patients in complete remission

Table 1 shows the basic characteristics of each group. As expected, the group with diabetes had a higher mean age and higher rates of hypertension, dyslipidemia, and smoking than the other groups. The majority of patients in remission from HL had stage IIa disease at diagnosis (Figure 1). Sixteen were treated with the ABVD protocol and one with the BEACOPP protocol. Four patients (24%) received radiotherapy.

**Table 1:** Clinical characteristics of the patients with HL in remission and the three control groups

Characteristic	HL in remission (N = 17)	Acute HL (N = 4)	Diabetes (N = 28)	Healthy (N = 16)	*P* value HL in remission vs diabetes	*P* value HL in remission vs healthy
Age (yr), average±SD (median)	33 ± 6 (33)	29 ± 5 (30)	61 ± 8 (69)	32 ± 4 (32)	<0.001	0.9
Gender (M, %)	8 (47.1%)	2 (50%)	21 (75%)	8 (50%)	0.3	1.0
Hypertension, n(%)	0	0	20 (71%)	0	<0.001	1.0
Smoking, n(%)	6 (35%)	0	18 (64%)	1 (6%)	0.2	0.3
Hyperlipidemia n(%)	8 (25%)	2 (50%)	27 (93%)	0	<0.001	0.3

HL, Hodgkin's lymphoma.

**Figure 2:**
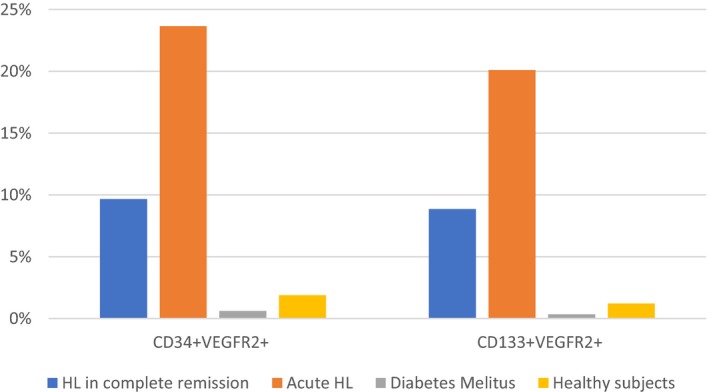
Circulating levels of EPC markers CD34(+)/VEGFR2+ and CD133(+)/VEGFR2+ by study groups

**Figure 3:**
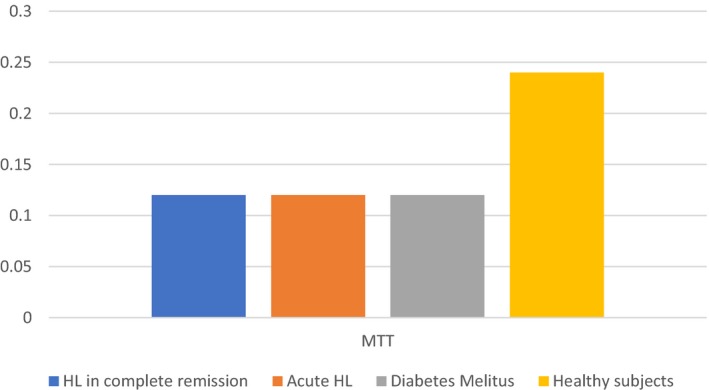
MTT scores by study groups

**Figure 4:**
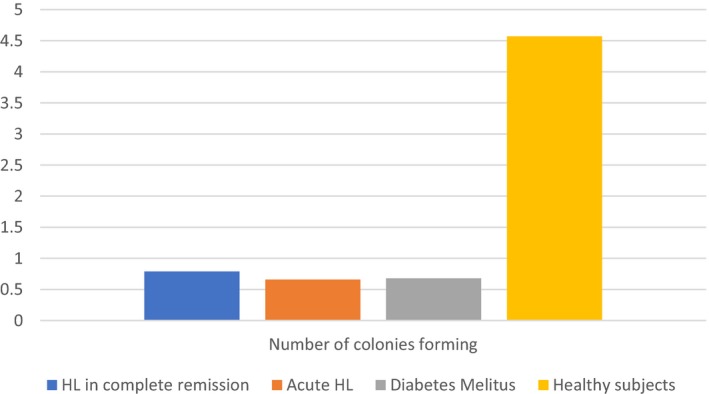
Number of CFUs by study groups

Mean levels of EPC markers CD34(+)/VEGFR2(+) and CD133(+)/VEGFR2(+), indicating the rate of circulating EPCs, were significantly higher in the patients with newly diagnosed untreated HL than in the patients with HL in remission (*P* = 0.03 and *P* = 0.005, respectively, Figure 2). There was no significant difference between these groups in the number of CFUs or MTT score, indicating cell viability (Figures 3 and 4). Patients in remission from HL had significantly higher levels of EPC markers than patients with diabetes (*P* = 0.011 and *P* < 0.001, respectively, Figure 2), with no difference in MTT score or number of CFUs. Comparison of the patients in remission and the healthy group yielded significantly higher levels of EPC markers in the remitted HL group (*P* = 0.08 and *P* = 0.003, respectively) in addition to a significantly lower MTT score (*P* = 0.005, Figure 3) and a lower CFU count (*P* < 0.001, Figure 4). Patients with diabetes had a lower MTT score (*P* = 0.01) and CFU count than the healthy subjects (*P < *0.001) (Figures 3 and 4), although their circulating EPC level was similar (Figure 2).

## 4 DISCUSSION

Endothelial injury is the cornerstone of atherosclerotic cardiovascular disease. Circulating EPCs have an essential role in the repair of vascular injury,^1^ and attenuated EPC function is characteristic of patients with significant cardiovascular risk factors, such as diabetes, hypertension, and hyperlipidemia.^4^, ^7^

The present study yielded a unique profile of EPCs in patients with HL in remission. The patients were relatively young and had no history of CAD and were considered cured of their lymphoproliferative disease. The EPCs were sampled at least 2 years after the therapy courses were completed, and none of the patients had overt anthracycline‐related cardiotoxicity. Nevertheless, they showed a mixed EPC pattern: The level of circulating EPCs was conspicuously increased relative to healthy controls, almost to the level of the patients with acute HL before onset of but EPC function (MTT score and capacity to form colonies) was compromised, similar to that in the diabetic patients who were at high risk of CAD.

In both groups of patients with HL, circulating EPC level was prominently increased alongside reduced EPC function compared to the healthy subjects. This finding cannot be interpreted as a consequence of the malignant illness, as unlike the acute HL patients, the patients with remitted HL had been disease‐free for at least 2 years. At the same time, it cannot be attributed to the impact of chemotherapy and radiation on cardiovascular toxicity, because unlike the remitted HL group, the newly diagnosed patients had not yet been treated. Hence, we assume a multifactorial effect of both the malignant disease and the chemotherapy to explain our observation. The malignancy induces the first peak in the circulating dysfunctional EPC level. The decline in EPC number reflects the remission; however, the chemotherapy seems inadequate to resume normalization in circulating EPC number and function and may as well contribute to the EPC dysfunction.

This study was not designed to examine the cardiovascular morbidity and mortality in patients with remitted HL. Further studies are needed to investigate potential long‐term clinical consequences of the abnormal EPC profile characterizing young adult patients (age 20‐50 years) without cardiac disease considered cured of HL at least 2 years after completing treatment, in addition to the need for closer longer‐term surveillance in these cases. Furthermore, in light of recent evidence that statins may help to normalize the function of circulating EPC,^27^, ^28^ our findings suggest that studies investigating the value of adding statins to the maintenance therapy of patients with HL in remission may be warranted.

Our results albeit significant were found in small size groups. The study was not designed to examine the cardiovascular morbidity in our patients with remitted HL. Further research is warranted to investigate whether the sustained abnormal EPC profile characterizing the patients in remission has long‐term clinical consequences for an adverse outcome.

## CONFLICT OF INTEREST

None declared.
